# The effect of housing on the mental health of older people: the impact of lifetime housing history in Whitehall II

**DOI:** 10.1186/1471-2458-11-682

**Published:** 2011-09-02

**Authors:** Philippa L Howden-Chapman, Tarani Chandola, Mai Stafford, Michael Marmot

**Affiliations:** 1 He Kainga Oranga/Housing and Health Research Programme, University of Otago, 23a Mein St, Wellington, 6021, New Zealand; 2 The Cathie Marsh Centre for Census and Survey Research, University of Manchester, UK; 3 MRC Unit for Lifelong Health and Ageing, University College London, UK; 4 Department of Epidemiology and Public Health, University College, London, UK

**Keywords:** Housing, mental health, financial problems, older people

## Abstract

**Background:**

This study describes differences in trajectories of self-reported mental health in an ageing cohort, according to their housing, while controlling for confounders.

**Methods:**

The General Health Questionnaire was measured on six occasions as part of Whitehall II cohort study of office-based British civil servants (1985-2009); 10,308 men and women aged 35-55 at baseline.

**Results:**

Home-ownership was the predominant tenure at baseline and increased over the life-course, but the social gradient remained. In the bivariate analysis, by phase nine, renters had higher (poorer mental health) GHQ scores (55.48) than owner occupiers (51.98). Those who reported difficulty paying bills or problems with housing had higher GHQ scores at baseline (financial difficulties 57.70 vs 54.34; house problems 58.06 vs 53.99) and this relative difference increased by phase nine (financial difficulties 59.64 vs 51.67; house problems 56.68 vs 51.22). In multivariate models, the relative differences in GHQ scores by tenure increased with age, but were no longer significant after adjusting for confounders. Whereas GHQ scores for those with housing problems and financial difficulties were still significantly higher as participants grew older.

**Conclusion:**

The social gradient in the effect of home ownership on mental health, which is evident at baseline, diminishes as people get older, whereas housing quality and financial problems become relatively more important in explaining older people's health. Inequalities in housing quality and ability to deal with household financial problems will become increasingly important mental health issues as the population ages.

## Background

Self-reported mental health generally improves by early old age, but social class differences in anxiety and depression increase with age [1]. In the Whitehall II study, social inequalities in both self-reported mental health and general health increased in early old age, as the rate of improvement in mental health was less for those in the lower employment grades [2]. Using a framework derived from the social determinants of health, we summarise the direct and indirect impact of housing patterns on health inequalities [3]. We then analyse the specific roles of housing tenure and quality, as well as financial security over the life course, in explaining the pattern of improving mental health, but increasing mental health inequalities in the Whitehall II Study. We conclude by discussing which public policies could reduce mental health inequalities in older people.

### Housing patterns and health inequalities

Studies of patterns of health inequalities in older age groups are primarily focused on 'lifestyle' rather than structural variables and largely ignore possible explanatory variables such as housing,[4] despite strong evidence linking housing tenure to adult health in various longitudinal [5,6] and cohort studies [7,8]. Housing costs, including fuel use, rent or mortgages, maintenance and repairs, are a significant component of the minimum income for healthy living required by older people [9] and those who are home-owners may be mortgage-free by the time they retire. Time-use surveys consistently show that older people spend more than 90% of their time indoors, mostly in their homes, [10] so that the indoor home environment is their most significant environmental exposure, as well as being the place that they have most contact with their families or friends.

Generally, housing affects people's health at several levels [11]. Housing tenure is a structural variable; houses are usually the largest capital asset owned by families and this wealth can be used to generate a stream of income, in addition to salary, wages and benefits. In Britain, wealth is highest for those close to retirement, but inequalities are pronounced and magnified by differential access to pensions [12,13].

Those who rent, whether from a private landlord or a social housing agency, are likely to be poorer, although in some cases they may be trading off more income for less wealth [14]. There is also a possible cultural effect of tenure choices on mental health. In England, people generally aspire to home ownership and renting is seen as a temporary measure. Renting permanently is more unusual, or may be seen as a sign of failure (particularly renting in the public sector). However, in some European countries, renting is just another housing option. Nonetheless, there is cross-cultural evidence that people who own their own houses are in better health than people who rent their houses, even controlling for income [15].

Home-ownership seems to confer both psychological and material advantages on owner occupants, [16-18] although a recent systematic review concludes the evidence is not strong [19]. Psychologically, owning a home rather than renting seems to confer greater autonomy and social status [20]; what economists call 'positional goods'. Houses that are owned are generally in better condition than rented accommodation. Rental housing is generally of poorer quality and more insecure. Leases, though they vary from country to country, do not give the same security to tenants as a house title gives to an owner [21]. However, this is not a static situation, in part because the housing market is such a pivotal part of the general economy and in an economic recession, home-owners, who bought in a boom, may be left with negative equity in their houses [22,23]. In this case, home-ownership may be *less *secure than rental housing, particularly if the home-owner is made unemployed or becomes chronically ill. Mortgage payment commitments and the costs of maintaining housing can be stressful and the quality of housing that can be afforded on reduced incomes may be less health promoting than rental housing that can be afforded for the same expenditure [17,24].

Housing quality is also an intervening variable between SES and health in producing social inequalities in health. Cold, damp, mouldy housing affects people's health and well-being, as well as their use of health services [25,26]. Housing conforms to the inverse care law first identified in health care in Britain [27]. Colder and windier parts of the UK have poorer housing, which is associated with reduced lung function, as well as raised diastolic and systolic blood pressure [28]. People living in cold homes are more likely to have poorer mental health [29]. Those in single-person households tend to have higher living costs and are more likely to suffer from fuel poverty, i.e. they need to spend more than 10% of their income on household energy to maintain indoor temperatures to an adequate level [30,31]. Fuel poverty has been exacerbated by the retail price of domestic fuel increasing by 91% between 2002 and 2009 [13]. Experiencing financial difficulties in general may well capture fuel poverty in particular.

Le Grand has argued that housing is no different from any other good in a capitalist society and should be considered fundamentally in monetary terms [32]. The contrary view is that the ontological security provided by housing is high on the hierarchy of needs and confers a unique range of services. Analysis of the Joseph Rowntree Foundation Poverty and Social Exclusion Survey showed that poor renters were significantly more likely to be dissatisfied with their neighbourhoods (10% vs 4%), but poor home-owners were more likely to report a structural problem with their house, such as a leaky roof (13% vs 4%) [33]. Poor home-owners were more likely to report poor mental health than renters, but this result was not significant.

#### Housing and mental health

Physical and mental health are clearly interconnected, but research on housing and mental health is particularly underdeveloped [34]. There is sufficient evidence to suggest that the type and quality of housing affects psychosocial processes, which in turn can affect mental health in a variety of ways, such as identity and self-esteem, anxiety about structural hazards, worry and lack of control over maintenance and fear of neighbourhood crime [35,36]. A cross-sectional survey of adults in two electoral wards in one northern London borough, which had independent measures of the built environment and controlled for SES and structural problems in the houses, found a significant increase in cases of depression in those living in newer housing where access was from a common balcony [37].

While around 16% of the English Longitudinal Survey of Ageing participants report housing problems, there have as yet been few analyses that link housing problems with any health measures [13].

#### Methodological issues

While most of these data come from cross-sectional studies, in this paper, we use data from the longitudinal Whitehall II study, which have many advantages, but some disadvantages. People were recruited in middle years and there has been a high retention rate; the oldest cohort member was born in 1930, the youngest in 1952. While basically a London cohort, there is still considerable variation in housing quality, as in the UK as a whole [38].

On the other hand, the cohort is clearly skewed towards those who are employed in higher socio-economic positions and, as a consequence, there is a higher rate of home ownership than in the population as a whole, although there is still heterogeneity.

We explore the relationship between housing tenure, housing quality and household financial security over the adult life course on the mental health of older people, who are approaching retirement age or retired. Our hypothesis is that, controlling for other confounding factors:

1. Older people, who own their house, have better housing quality and fewer financial problems have fewer mental health problems than those people who rent their house.

2. The effect of these housing factors on mental health increases as people age.

## Methods

Data: The Whitehall II study is an ongoing longitudinal study of 10,308 male and female civil servants (initially aged 35 to 55) based in London and set up in 1985. (Whitehall II cohort profile http://ije.oxfordjournals.org/cgi/reprint/dyh372v1.pdf) The first (1985-88), third (1991-93 N = 8637), seventh (2002-2004 N = 6914) and ninth (2007-09 N = 6762) phases of the study were analysed. At the ninth phase, participants ranged in age from 55 to 80 years old; 7.3% are still employed in the civil service, although a further 13% are still in employment elsewhere. Their last known civil service employment grade is used for those who have left the civil service. 'Low grade' civil servants are Clerical and Office Support staff, as opposed to those who are Executive Officers and above.

### Variables

Outcome: The General Health Questionnaire (GHQ-30) is a 30-item questionnaire that measures minor psychiatric morbidity [39]. This was asked at all the phases of the study. The Likert scale scores were coded so that responses ranged from 1 (good health) to 4 (poor health). These scores were summed up to give a continuous score ranging from 4 to 120, with a normal distribution. A continuous GHQ score was chosen over a measure of GHQ caseness in order to describe changes and trajectories in mental health more accurately. The GHQ questionnaire was asked at all phases of the study.

### Exposures

Housing tenure was phase varying: Respondents were asked if the accommodation they lived in was owned or rented at each phase in this analysis. Rental accommodation was further split into those renting from local authority or housing association vs. renting privately. However, the numbers renting privately were too small to analyse as a separate category.

Housing quality was measured by a single question on '*To what extent do you have problems with your housing, e.g. 'too small, repairs, damp*', with responses ranging from *'very great problems' *to *'very little'*. These responses were categorised into a binary variable- *'some to very great problems' *vs. *'very little to slight problems'*. This question was asked at each phase in this analysis, except at phase 9. So responses at phase 8 of the study on housing quality were used to replace the missing phase 9 housing quality variable.

Household financial problems was measured by a single question '*To what extent do you have difficulty paying bills'*, with responses ranging from *'very great problems' *to '*very little'*. These responses were categorised into a binary variable- *'some to very great difficulty' *vs *'very little to slight difficulty'*. This question was asked at each phase in this analysis.

### Confounders

The confounders analysed in this study were gender (phase-invariant) and other phase-varying covariates including age in years occupational grade, retirement status, smoking status, alcohol consumption above recommended limits and marital status. Occupational grade, marital status, smoking, alcohol consumption and gender are potential socioeconomic confounders of the association between housing and health, while retirement status is a potential confounder of any factor associated with changes in mental health.

Ethical approval was given by University College London Medical School Committee on the Ethics of Human Research.

### Analysis

We first examined the bivariate cross-sectional associations of the housing exposures with GHQ-30 and other confounders at phases 1 and 9.

We then analysed GHQ-30 longitudinally using repeated measures (over four cross-sectional phases) multiple (linear) regression models, looking at the relative contribution of each of the housing variables-housing tenure, financial problems, and housing problems-on GHQ-30 mental health, while controlling for other confounding variables. Three nested multivariate models were compared. The most general model estimated the effect of each housing variable separately on GHQ-30 scores, adjusted for age and gender. The next model additionally included all the three housing variables together and their interactions with age. The final model additionally included all the confounding variables and their interaction with age. A quadratic function of age and interactions with the housing variables was initially included in all the models. However, this quadratic term of age and related interactions did not significantly reduce the deviance of the final model and so were excluded from the models shown in the results below.

A repeated measures multilevel model with phases (level 1) nested within respondents (level 2) was specified. A random effect of age (at level 2) was also specified in all the models to take account of heterogeneity in the effect of age on GHQ-30. For the final model, there are four units at level 1 (denoting the phase of the study) and 9846 units at level 2 (corresponding to most of the participants at baseline).

## Results

### Bivariate analyses

Housing tenure in the Whitehall II Study is skewed towards home-ownership and became more so over the life-course. At phase one (1985-88), when the participants were aged between 35 and 55, 92% of these respondents owned, partly or fully, a house and 8% rented. At phase 9, home ownership increased to around 96% of the sample. The social gradient in home ownership remained over the life course. At phase one, only 16% of all owner occupiers had low grade civil service jobs. By phase 9, this had reduced to 8%.

At phase one, there was not much difference in GHQ scores between those who were renting and home owners. However, by phase nine, those in rented housing had higher (poorer) GHQ scores (55.48) than owner occupiers (51.98). In terms of difficulty paying bills and problems with housing, those who reported some problems in either domain had higher (poorer) GHQ scores at phase one (for difficulty paying bills 57.7 vs 54.34 and for problems with house 58.06 vs 53.99), and this difference increased by phase nine (for difficulty paying bills 59.64 vs 51.67 and for problems with house 56.68 vs 51.22). At phase one of the study, the questions on housing problems and financial difficulties were not asked in all versions of the questionnaire, resulting in significantly lower proportions of responses. However, there was no selection of participants in terms of who received a particular version of the baseline questionnaire, suggesting that such non-response is not likely to bias the results.

### Multivariate analyses

We analysed a regression model with repeated measures of GHQ (continuous score) as the outcome variable. The independent variables included in Model 1 were age, period (entered as a dummy variable), housing variables separately, as well as the interaction of each housing variable separately with age as an explanatory variable, and specifying a random effect of age (see Table 2). Living in a rented house increased GHQ scores by 0.71. Furthermore, this increase in GHQ score grew larger as the participants grew older. We observed a similar, although larger effect of having some difficulty paying bills (this increased GHQ scores by 4.07), as well as problems with house (this increases GHQ scores by 3.31). The effect of both these problems/difficulties on increasing GHQ scores increased with age.

**Table 1:** Cross sectional associations of housing tenure and main variables in the analyses at the baseline and last phases of the Whitehall II study

	**Housing tenure**				**Difficulty paying bills**				**Problems with house**			
	
**Phase 1 (1985-88)**	**Owner-occupier**		**Rented housing**		**Slight/no difficulty**		**Some/great difficulty**		**Slight/no problems**		**Some/great problems**	
	**Mean or %**	***N***	**Mean or %**	***N***	**Mean or %**	***N***	**Mean or %**	***N***	**Mean or %**	***N***	**Mean or %**	***N***
% men	70.6%	8998	40.8%	1228	67.1%	6003	68.8%	1649	68.0%	5629	66.2%	2025
GHQ	55.05	8986	55.43	1220	54.34	6002	57.70	1648	53.99	5628	58.06	2024
Age	44.28	8998	45.62	1228	44.70	6003	43.91	1649	44.80	5629	43.80	2025
% low grade	16.4%	8998	67.3%	1228	22.2%	6003	29.0%	1649	23.0%	5629	25.3%	2025
% in rented housing	0.0%	8998	100.0%	1228	11.0%	5969	15.5%	1636	9.7%	5594	18.1%	2014
% some to great difficulty paying bills	20.7%	6697	27.9%	908	0.0%	6003	100.0%	1649	15.3%	5622	38.9%	2025
% with some to great housing problems	24.6%	6701	40.2%	907	20.6%	5999	47.8%	1648	0.0%	5629	100.0%	2025
% retired	0.0%	8998	0.0%	1228	0.0%	6003	0.0%	1649	0.0%	5629	0.0%	2025
% without cohabiting partner	21.4%	8967	56.9%	1223	25.7%	5983	24.9%	1633	23.4%	5606	31.2%	2011
**Phase 9 (2008-09)**	Owner-occupier		Rented housing		Slight/no difficulty		Some/great difficulty		Slight/no problems		Some/great problems	
	Mean or %	*N*	Mean or %	*N*	Mean or %	*N*	Mean or %	*N*	Mean or %	*N*	Mean or %	*N*
% men	71.8%	6270	52.1%	284	71.8%	6144	57.0%	407	71.6%	5771	65.3%	1023
GHQ	51.98	6267	55.48	284	51.67	6142	59.64	407	51.22	5351	56.68	915
Age	65.92	6270	66.37	284	65.97	6144	65.27	407	66.12	5415	65.45	935
% low grade	8.3%	2380	37.6%	117	8.4%	2333	26.1%	161	8.6%	2014	13.9%	359
% in rented housing	0.0%	6270	100.0%	284	3.7%	6103	13.8%	400	3.5%	5308	8.0%	900
% some to great difficulty paying bills	5.5%	6223	19.6%	280	0.0%	6144	100.0%	407	4.6%	5306	13.7%	901
% with some to great housing problems	13.9%	5949	27.8%	259	13.3%	5842	33.7%	365	0.0%	5771	100.0%	1023
% retired	68.4%	6007	68.7%	262	68.9%	5884	59.8%	378	69.0%	5202	67.5%	874
% without cohabiting partner	22.8%	6186	61.8%	272	23.7%	6055	37.4%	398	22.6%	5343	34.0%	912

**Table 2:** Repeated measures regression of GHQ by housing variables in the Whitehall II study, adjusted for potential confounders

	Model 1	Model 2	Model 3
*Predicted GHQ*	54.45**	53.92**	56.36**
*Age (years)*	-0.12**	-0.13**	-0.18**
*Housing tenure*			
Owner-occupier	ref.	ref.	ref.
Rented housing	0.71**	0.25	-0.10
Housing tenure*age	0.09**	0.06*	0.02
*Predicted GHQ*	54.53**	53.92**	56.36**
*Age (years)*	-0.12**	-0.13**	-0.18**
*Difficulty paying bills*			
Slight to no problems	ref.	ref.	ref.
Some to great problems	4.07**	3.54**	3.47**
Bill difficulty*age	0.12**	0.12**	0.09**
*Predicted GHQ*	54.45**	53.92**	56.36**
*Age (years)*	-0.11**	-0.13**	-0.18**
*Problems with house*			
Slight to no problems	ref.	ref.	ref.
Some to great problems	3.31**	2.90**	2.98**
House problems*age	0.04**	0.04**	0.04*

Model 1: Multiple regression model with GHQ (repeated measures) as outcome and age, gender, period, housing variables (separately) and interaction of each housing variable (separately) with age as explanatory variables; random effect of age specified.

Model 2: includes Model 1 and all housing variables together and their interactions with age

Model 3: includes Model 2 and grade and retired status and their interactions with age

* p < 0.05

** p < 0.01

In Model 2, also shown in Table 2, where all the housing variables are included at the same time, as well as their interaction with age, the results were similar, although the effect of housing tenure decreases. Model 3 includes Model 2 as well as possible confounders, cohabitation status, employment grade and retirement status and their interaction with age. Housing tenure no longer had a significant independent effect, whereas housing problems (increased GHQ score by 3.11) and financial difficulties (increased GHQ score by 3.40) retained their explanatory significance and increased over time.

**Figure 1:**
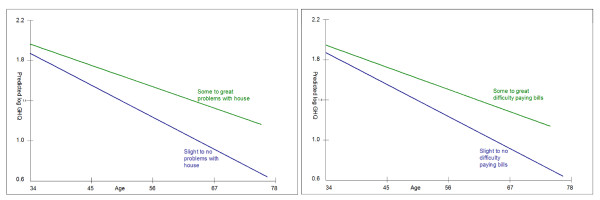
**Trajectories of log GHQ-30 with age by problems with house, and by difficulty paying bills**. Overall, mental health improves in older age groups (GHQ score reduces), but it improves less if people have 'some' or 'great' problems with their house and even less if they have 'some' or 'great difficulty paying bills, compared to those with fewer difficulties in these areas. Controlling for possible confounders (cohabitation status, employment grade, retirement status and their interaction with age, estimated from Model 3 in Table 2) this figure shows growing relative inequalities by age, related to housing problems and difficulties paying bills. Housing tenure was not significant.

These effects are shown in graph form in Figure 1. They show growing inequalities by age, by experiencing problems with housing and by difficulty paying bills, estimated from Model 3 in Table 2.

## Discussion

This relative importance of housing and financial security on health increases during working life and retirement. In the Whitehall II cohort, the mental health of people who owned their own houses, unlike those who rented their houses, consistently improved over their working life and continued during their retirement. This cumulative impact of housing tenure on the inequalities in mental health in older people increased when we took into account not only this structural factor, but also intervening factors such as housing quality and financial problems. But when we controlled for confounding variables, housing tenure no longer had a significant independent effect, whereas housing quality and financial difficulties retained their explanatory significance.

The finding that the quality of housing and financial security are more important explanatory factors in explaining the mental health of older people than housing tenure is supported by other studies. Experiencing financial difficulties at baseline was the only predictor in new episodes of depression in the General Psychiatric Morbidity Survey [40] and in the British Household Panel Survey, which adjusted for more objective measures of standard of living, such as occupational level [41].

The explanation for how "the social becomes biological" is likely to have many strands [42].(p.48) Housing is part of the network of health resources that can either promote health over the life-course or increase susceptibility to illness and disease [17]. However, the quality of housing is particularly important to health at older ages, because susceptibility to low temperature increases with age and older people are exposed more than other age groups to the indoor home environment. Moreover data from the first five waves of the British Household Panel Survey, which looked at residential mobility for those over 55, found relatively few older people moved house [43]. In what might be another case of the inverse care law, older people on low incomes may also lack the funds to maintain and repair their homes, or afford the co-payments to take up public funds to improve their houses through retrofitted insulation and boilers and to pay their heating bills.

In policy terms, housing remains an important way of improving older people's health. Successive governments have encouraged home ownership through various tax subsidies and Right-To-Buy schemes. However, the relationship between housing tenure, quality, financial status and health may not always be direct. The Whitehall II study enables us to look at the direct and indirect interrelationship between the broad aspects of the determinants of health (housing, employment, financial problems and so on) to show that, after controlling for intervening variables such as employment grade, financial problems and housing quality, housing tenure is no longer a significant explanation of mental health in retirement. These results suggest, that as in the Burrows' study, owning a home in poor condition, without the financial resources to remediate it, may be a health burden for the owner occupier [33].

There are however, some methodological caveats to our results: both the independent variables (tenure, housing quality and financial problems) and the dependent variable (psychological well-being) are based on self-report, so that some of the covariance between housing quality and mental health might be created by the overlap in method. While self-perceptions are generally powerful predictors of health, the self-perception of housing quality used in this analysis was non-specific; future studies of the relative impact of housing tenure and housing quality would be strengthened by having independent measures of housing quality. Another limitation is the low proportion of participants living in rented housing, well below the average of around 22% of the population in this age group in the 2001 England and Wales census. This reflects the socioeconomically advantaged nature of the sample, namely those employed in the civil service. However, the Whitehall II study was never designed to be representative of the British population. Instead, its strength lies in discovering aetiological relationships on the social determinants of health. An association between housing problems and health in this advantaged sample suggests that this association would be even stronger in the general population with a greater proportion of socioeconomically disadvantaged groups.

Selection biases due to missing data are a problem inherent in all longitudinal studies, especially so in ageing studies. With over 1300 deaths in the cohort up to the ninth phase of the study, those remaining in the study are healthier on average than non-participants and are also more socioeconomically advantaged. However, this pattern of non-response would only affect the results presented here if the association between housing and mental health differed between those remaining in the study and non-participants at later phases of the study. Previous analysis of non-participation in the Whitehall II cohort has shown that the association between non-response and mortality does not differ by socioeconomic group [44]. This suggests that the pattern of non-response in this analysis may not have biased the results.

In this paper we have looked at only one health outcome, but it is biologically plausible that a number of other health symptoms, such as respiratory and coronary symptoms could also be affected by housing and the indoor environment, as poor housing has been related to cardiovascular disease [28]. The London-focus of the Whitehall II study also means that we have smaller variation in housing quality than if it were a national cohort. All these factors may under-estimate the relationship between housing and the mental health of older people.

## Conclusion

In common with most populations, those who owned a house in the Whitehall II study had better mental health and this effect increased as they aged. However, housing quality and financial problems became more important in explaining older people's health in the latest wave of the cohort than tenure. This study has highlighted that inequalities in housing quality together with a household's ability to deal with financial problems have a small, but significant effect on mental health. Housing quality and financial security, exemplified by the differential availability of pensions, will have increasing importance for mental health as the population ages.

PLHC was a Balzan Fellow at the Department of Epidemiology and Public Health at University College London and is Professor of Public Health at the University of Otago, Wellington, New Zealand, TC is Professor of Sociology, Manchester University, MS is a senior scientist at the MRC Unit for Lifelong Health and Ageing. MM is professor at Department of Epidemiology and Public Health, University College London.

## Competing interests

The authors declare that they have no competing interests.

## Authors' contributions

PLHC wrote the first draft, suggested analytical strategies, and is guarantor. TC carried out the analysis and contributed to subsequent drafts of the paper by writing sections. MS and MM contributed to subsequent drafts of the paper. All authors have read and approved the final manuscript.

## Pre-publication history

The pre-publication history for this paper can be accessed here:

http://www.biomedcentral.com/1471-2458/11/682/prepub
